# Caspase-2 resides in the mitochondria and mediates apoptosis directly from the mitochondrial compartment

**DOI:** 10.1038/cddiscovery.2016.5

**Published:** 2016-02-15

**Authors:** M Lopez-Cruzan, R Sharma, M Tiwari, S Karbach, D Holstein, C R Martin, J D Lechleiter, B Herman

**Affiliations:** 1 Department of Cellular and Structural Biology, The University of Texas Health Science Center at San Antonio , 7703 Floyd Curl Drive, MED 238D.2 , San Antonio, TX 78229-3900, USA; 2 Center for Biomedical Neuroscience, University of Texas Health Science Center at San Antonio, San Antonio, TX 78229, USA

## Abstract

Caspase-2 plays an important role in apoptosis induced by several stimuli, including oxidative stress. However, the subcellular localization of caspase-2, particularly its presence in the mitochondria, is unclear. It is also not known if cytosolic caspase-2 translocates to the mitochondria to trigger the intrinsic pathway of apoptosis or if caspase-2 is constitutively present in the mitochondria that then selectively mediates this apoptotic effect. Here, we demonstrate the presence of caspase-2 in purified mitochondrial fractions from *in vitro*-cultured cells and in liver hepatocytes using immunoblots and confocal microscopy. We show that mitochondrial caspase-2 is functionally active by performing fluorescence resonance energy transfer analyses using a mitochondrially targeted substrate flanked by donor and acceptor fluorophores. Cell-free apoptotic assays involving recombination of nuclear, cytosolic and mitochondrial fractions from the livers of wild type and *Casp2*^*−/−*^ mice clearly point to a direct functional role for mitochondrial caspase-2 in apoptosis. Furthermore, cytochrome *c* release from *Casp2*^*−/−*^ cells is decreased as compared with controls upon treatment with agents inducing mitochondrial dysfunction. Finally, we show that *Casp2*^*−/−*^ primary skin fibroblasts are protected from oxidants that target the mitochondrial electron transport chain. Taken together, our results demonstrate that caspase-2 exists in the mitochondria and that it is essential for mitochondrial oxidative stress-induced apoptosis.

## Introduction

Since its discovery,^1,2^ caspase-2, the most conserved cysteine aspartate protease among species, has been suggested to play key roles in apoptosis induced by various stimuli, including DNA damage, mitotic catastrophe, immunological defenses, trophic factor deprivation, broad spectrum kinase inhibition, *β*-amyloid signaling, oogenesis, tumorigenesis and mitochondrial dysfunction-mediated oxidative stress.^3–7^ In addition, several non-apoptotic roles for this caspase have also been proposed, such as the activation of NF-*κ*B and p38^MAPK^^8^ and translational upregulation of p21 expression.^9^ For a caspase with so many functions, it is surprising that data regarding its subcellular localization have been equivocal.^10^

A major point of inconsistency is its presence in the mitochondria. In addition to being localized to the cytosol, nucleus, endoplasmic reticulum (ER) and Golgi, caspase-2 has also been detected in the mitochondrial compartment using immunodetection approaches, such as western blots and immunostaining in cells;^11–18^ however, this finding is controversial because other groups have been unable to identify caspase-2 in this compartment.^15,19^ Additionally, it is unclear if the initial activation of caspase-2 occurs always in the cytosol or if caspase-2 activation can initiate at other sites, such as the nucleus and mitochondria. It is thought that caspase-2 cleaves the proapoptotic protein, Bid, that induces mitochondrial outer membrane permeabilization (MOMP), releasing cytochrome *c* and thereby activating caspase-3 via the apoptosome.^5,6,20,21^ These data suggest a role for the caspase-2 upstream of mitochondrial pathway. In contrast, processing of caspase-2 is suppressed in cells deficient for caspase-9 or Apaf-1,^22,23^ suggesting that caspase-2 functions downstream of MOMP. Recent studies using a bimolecular fluorescence complementation method suggest that caspase-2 activation occurs mainly in the cytoplasm;^10,24^ this study used heat shock and cytoskeletal disruptors as stimuli to activate caspase-2.

While it is possible that these contrasting observations are specific to certain stimuli or cell types, a major reason behind these equivocal results is also the lack of caspase-2-specific reagents as well as the use of indirect techniques to assess the role of caspase-2. For example, studies using cleavage of caspase-2 as a marker for its activation can be misleading because caspase-2 is activated by proximity-based dimerization and does not need to be cleaved for function.^25^ Similarly, data acquired via caspase activity assays using VDVAD-based substrates aloneare ambiguous as they are also cleaved, albeit to a lesser extent, by other caspases, including caspase-3.^26^ Finally, studies involving fusion of GFP to caspases^27^ to determine localization may also be erroneous, as has been shown recently for pro-caspase-1.^28^

Because our data indicated that caspase-2 plays a crucial role in mitochondrial oxidant-induced apoptosis and that lack of caspase-2 decreases apoptosis under these conditions,^29^ we hypothesized that mitochondrial caspase-2 could play an important role in apoptosis induced by dysfunction in that organelle. In the present study, we aimed to determine whether caspase-2 is localized and activated *in situ* in the mitochondria using several methods including fluorescence resonance energy transfer (FRET) and cell-free apoptosis involving recombination of mitochondria from wild type (WT) or *Casp2*^*−/−*^ mice with other organelles. Cumulatively, our results point to the presence of caspase-2 in mitochondria that is required for apoptosis.

## Results

### A subset of Casp2 localizes to the mitochondria

**Figure 1:**
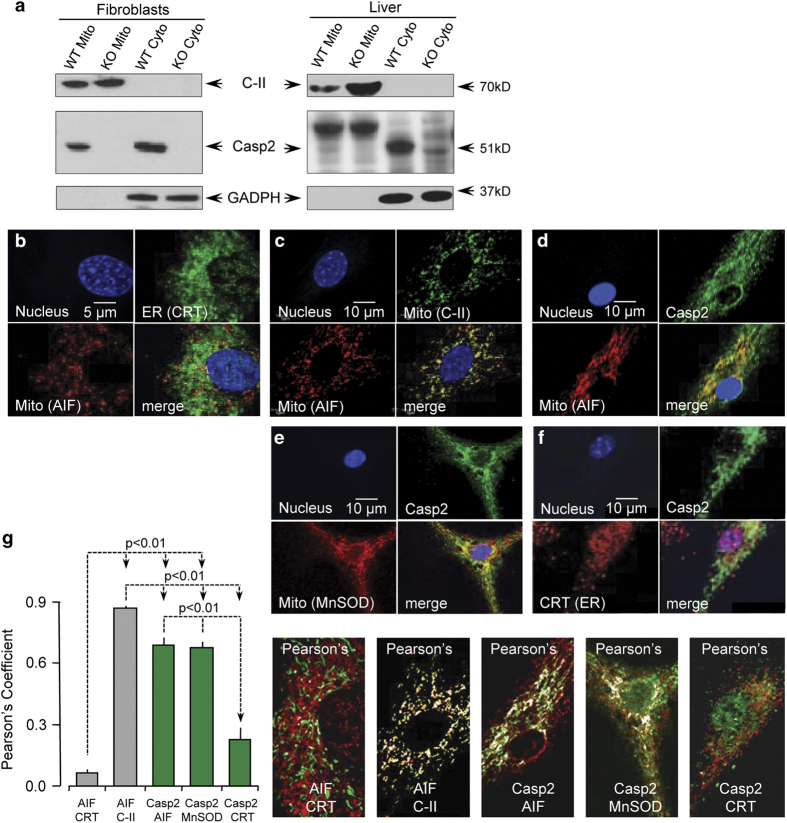
Caspase-2 localizes to the mitochondria. (**a**) Immunoblots for caspase-2 in purified mitochondrial extracts sourced from mouse embryonic fibroblasts (MEFs) or liver. Complex II (C-II) was used as a marker for mitochondria (mito) and also acted as a loading control. GAPDH was used as a loading control for cytoplasmic (cyto) extracts. WT=wild type and KO= *Casp2*^*−/−*^. (**b**) Primary MEFs were co-immunostained for AIF (red) and calreticulin (green), (**c**) AIF (red) and C-II (green), (**d**) AIF (red) and Casp2 (green), (**e**) MnSOD (red) and Casp2 (green), and (**f**) Calreticulin (CRT; red) and Casp2 (green). Nuclei in all images were stained with Hoechst 33342 (blue). (**g**) Pearson’s correlation coefficient based on a total of 12 cells per sample from three independent experiments is shown along with representative images.

To determine whether endogenous caspase-2 localizes to the mitochondria, we isolated mitochondrial and cytosolic fractions by differential centrifugation from naturally transformed WT and *Casp2*^*−/−*^ mouse embryonic fibroblasts (MEFs) and from age-matched WT and *Casp2*^*−/−*^ liver. We find caspase-2 expression in mitochondria of MEFs *in vitro* and in liver *in vivo* (Figure 1a). Purity of the mitochondrial and cytosolic fractions is indicated by the lack of GAPDH and complex II (C-II), respectively. We also co-immunostained primary WT MEFs with a monoclonal antibody towards caspase-2 and either mitochondrial proteins such as AIF, C-II and MnSOD or the ER such as calreticulin (CRT). Specificity of the anticaspase antibody was determined by lack of staining with *Casp2*^*−/−*^ MEFs (Supplementary Figure S1). Pearson’s correlation coefficient, which ranges in values between 0 (complete exclusion) and 1 (complete colocalization), indicates that a subset of caspase-2 resides in the mitochondrial compartment (Figures 1d, e and g), supporting our immunoblot data. In contrast, there was little overlap of caspase-2 with CRT, suggesting that very little caspase-2, if any, is present in the ER. Significant colocalization of mitochondrial proteins, AIF and C-II (Figure 1c), and very little overlap between mitochondrial and the ER proteins (AIF and CRT; Figure 1b) validate the antibodies used and our methods.

### Mitochondrial caspase-2 can trigger apoptosis in cell-free systems

**Figure 2:**
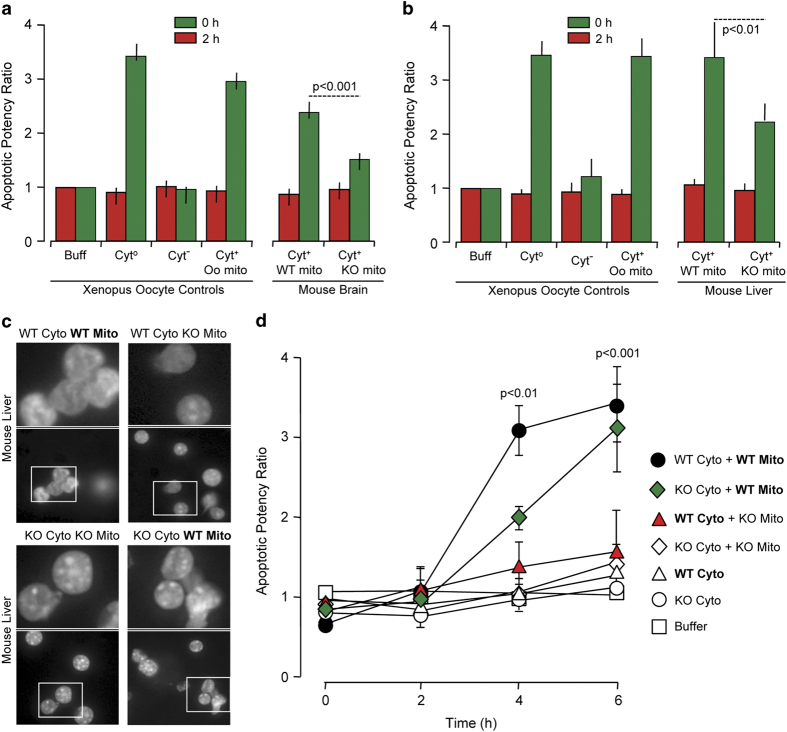
Mitochondrial caspase-2 can trigger apoptosis in cell-free systems. Rat liver nuclei and *Xenopus* oocyte cytosol were mixed with purified mitochondria from (**a**) WT or *Casp2*^*−/−*^ brain or (**b**) WT or *Casp2*^*−/−*^ liver and incubated at 37˚C for 4 h. Shown are the apoptotic potency ratios (calculated as the percent ratio of apoptotic nuclei in sample to buffer) from three independent experiments. (**c**) Representative images of liver nuclei mixed with cytoplasmic and mitochondrial fractions from either WT or *Casp2*^*−/−*^ mice at 4 h. Upper panels are magnified images of the insets indicated in the respective lower panels. Panels 1–4 contain WT cytoplasmic fraction whereas panels 5–8 contain *Casp2*^*−/−*^ cytoplasmic fraction. (**d**) Apoptotic potency ratios of the different combinations at the indicated time points.

Next, we wanted to determine whether mitochondrial caspase-2 is functionally active and can trigger apoptosis from the mitochondria. Since proteolytic cleavage of caspase-2 is not required for initial activation^25^ and since substrate-conjugated fluoromethylketone assays are not specific, we utilized a cell-free system containing mitochondria from either WT or *Casp2*^*−/−*^ mouse brain (Figure 2a) or liver (Figure 2b), *Xenopus* oocyte cytosol, and rat liver nuclei. In this reconstituted cell system, only the mitochondrial fraction and not the cytosolic or ER fractions triggers an apoptotic phenotype in nuclei such as fragmentation or condensation.^30,31^ As expected, *Xenopus* whole-cell lysate (control; either Cyt° or crude) induced high apoptotic nuclei numbers while cytosol alone (Cyt^−^ or pure) failed to induce this feature (Figure 2a). Also, as expected, reconstituted *Xenopus* cytosol with *Xenopus* mitochondria (Cyt^+^ Oo mito) generated a substantial increase in apoptotic nuclei. Similarly, reconstituted *Xenopus* cytosol with WT mouse brain mitochondria (Cyt^+^ WT mito) induced almost as many apoptotic nuclei as the reconstituted *Xenopus* preparation (Figure 2a and Supplementary Figure S2). However, when mitochondria from *Casp2*^*−/−*^ mouse brain was added (Cyt^+^ KO mito), the number of apoptotic nuclei was significantly lower at 4 h of incubation than that with the addition of the WT mitochondria. Similar results were obtained with *Casp2*^*−/−*^ and WT liver mitochondria (Figure 2b). The data also demonstrated a small residual amount of nuclear apoptosis even when caspase-2 was not present in the mitochondria, implying that an additional component in the oocyte cytosol could potentially act on mitochondrial proteins to induce nuclear apoptosis.

Caspase-2 is the most conserved caspase and *Xenopus* oocytes contain caspase-2. Therefore, to rule out any contribution of *Xenopus* caspase-2 in the above experiments, we isolated cytosol, mitochondria and nuclei from WT and *Casp2*^*−/−*^ liver and performed several mix-and-match combinations to validate our results using the above technique (Figures 2c and d). We reconstituted WT cytosol with WT mitochondria (WT cyto+WT mito), *Casp2*^*−/−*^ cytosol with WT mitochondria (KO cyto+WT mito), WT cytosol with *Casp2*^*−/−*^ mitochondria (WT cyto+KO mito), *Casp2*^*−/−*^ cytosol with *Casp2*^*−/−*^ mitochondria (KO cyto+KO mito), WT cytosol alone (WT cyto) and *Casp2*^*−/−*^ cytosol alone (KO cyto). Neither WT nor *Casp2*^*−/−*^ cytosol alone induced any apoptotic feature in the nuclei at the time points measured. The reconstituted WT system (WT cyto+WT mito) induced apoptotic features at 4 h that significantly increased at 6 h of incubation. Similarly, WT mitochondria reconstituted with *Casp2*^*−/−*^ cytosol progressively induced nuclear apoptosis, with numbers of apoptotic nuclei similar to the total reconstituted WT system at 6 h. The reconstituted *Casp2*^*−/−*^ system failed to induce apoptotic nuclei and most importantly, reconstituted samples that lacked caspase-2 in the mitochondria also failed to induce apoptotic nuclei despite the presence of caspase-2 in the cytosol (WT cyto+KO mito). Therefore, the presence of mitochondrial caspase-2 appears to be necessary for inducing nuclear apoptosis regardless of the presence or absence of cytosolic caspase-2.

### Mitochondrial caspase-2 induces cytochrome *c* release

**Figure 3:**
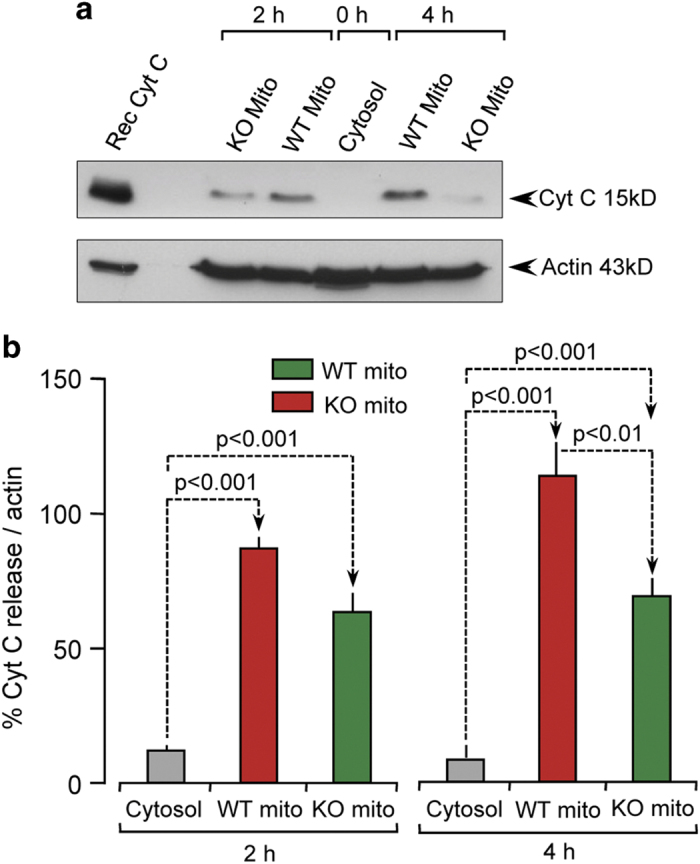
Mitochondrial caspase-2 induces cytochrome *c* release. *Xenopus* oocyte cytosol was mixed with purified mitochondria from either WT or *Casp2*^*−/−*^ brain, centrifuged to separate mitochondrial and cytosol fractions and immunoblotted for cytochrome *c* (cyt *c*). (**a**) Representative blot from three independent experiments; Rec denotes recombinant cyt *c* loaded as a positive control. (**b**) Densitometry analysis after normalization to actin.

Measuring nuclear apoptotic morphology only provides information pertaining to the end point of apoptosis. To determine whether mitochondrial caspase-2 was important in initiating apoptosis, we analyzed if caspase-2 could induce cytochrome *c* release from the mitochondria. We used the cell-free apoptosis system as described above; however, nuclei were not added. At 0, 2 and 4 h of reaction, we centrifuged the samples to separate the cytosol and mitochondrial fractions. The cytosol fraction from each reaction mixture was probed for cytochrome *c* using immunoblots (Figure 3). The amount of cytochrome *c* released into WT cytosol increased with increasing time. In contrast, the absence of caspase-2 resulted in significantly lower levels of cytochrome *c* release into the cytosol relative to the WT samples at both 2 and 4 h. *Xenopus* cytosol alone did not contain any cytochrome *c*.

### Oxidative stress induces mitochondrial caspase-2-like activity

**Figure 4:**
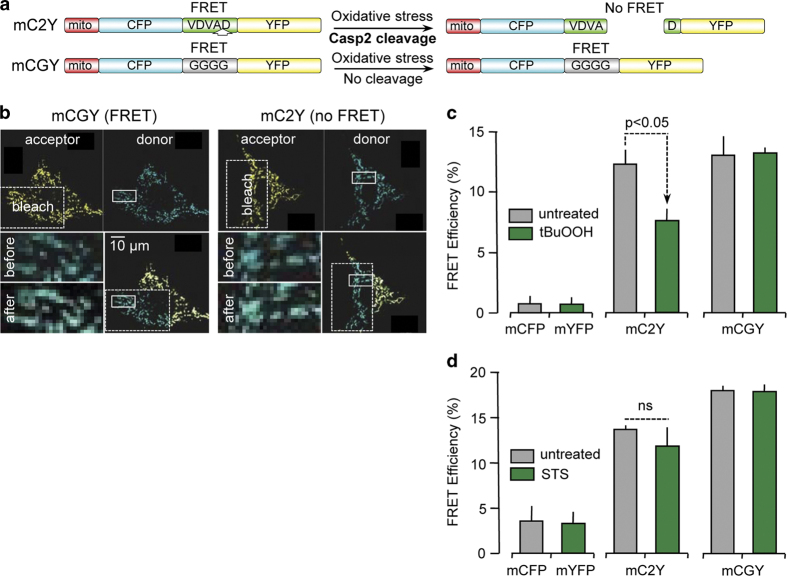
Oxidative stress induces mitochondrial caspase-2-like activity. NIH 3T3 cells were transfected with a (a) combination of mCFP and mYFP, or (b) mCGY, or (c) mC2Y and analyzed for FRET. (**a**) Cartoon showing the mechanistic basis of the mitochondrial-targeted constructs. Close proximity of CFP and YFP will result in FRET that is lost when mC2Y is cleaved by mitochondrial caspase-2 under conditions of oxidative stress. mCGY cannot be cleaved by caspase-2 and demonstrates FRET activity constitutively. (**b**) Representative FRET images of cells transfected with either mCGY or mC2Y in the presence of 100 *μ*M *tert*-butyl hydroperoxide (*t*-BuOOH). (**c**) Analysis of FRET efficiency after treatment with 100 *μ*M *t*-BuOOH for 1 h. (**d**) Analysis of FRET efficiency after treatment with 0.5 *μ*M staurosporine (STS) for 5 h. Data are representative of three independent experiments in which 5–10 fields were analyzed per cell and 5–10 cells were quantified per experiment.

We have previously shown that oxidative stress triggers caspase-2 activity.^29^ To discern if mitochondrial caspase-2 is also activated by oxidants and other treatments that induce apoptosis, we generated DNA constructs containing an N-terminal mitochondrial targeting sequence and the preferential caspase-2 substrate peptide, VDVAD, that was flanked by a fluorescent donor (ECFP; enhanced cyan fluorescence protein) and an acceptor (EYFP; enhanced yellow fluorescence protein) protein (mC2Y; Figure 4a). This construct allowed us to perform FRET techniques to measure real-time caspase-2 activity based upon cleavage of the substrate. In addition, we used the same backbone construct but replaced the preferential caspase-2 substrate with a string of glycine residues of the same length (mCGY). Since this construct cannot be cleaved by caspase-2, it functions as a positive control for FRET. The expression of the fluorescent protein constructs was found to be exclusively in the mitochondria (Supplementary Figure S3 and Figure 4b). As a negative control, we co-transfected mECFP and mEYFP together, both targeted to the mitochondria using the same mitochondrial targeting sequence (Supplementary Figure S4). Because NIH/3T3 mouse fibroblast cells are relatively easy to transfect, we used these cells in the experiments described here. In this assay, caspase-2 activity results in a decrease in FRET efficiency due to cleavage of the caspase-2 substrate sequence in the FRET reporter construct and consequent increase in the distance between the donor and acceptor fluorophores. Following transfection and expression of the reporter construct, cells were exposed to the mitochondrial oxidant *tert*-butyl hydroperoxide (100 *μ*M *t*-BuOOH; Figure 4c) or the general protein kinase inhibitor, staurosporine (0.5 *μ*M STS; Figure 4d). Optimal treatment times for *t*-BuOOH and STS were 1 and 5 h, respectively. For these reasons, we carried out two sets of positive and negative controls, one for *t*-BuOOH and one for STS, and plotted them in two different graphs with the same *Y*-axis range. STS did not provide any significant loss in FRET efficiency (Figure 4d), indicating no cleavage of mC2Y by caspase-2. In contrast, treatment of cells with *t*-BuOOH induced a significant decrease in FRET efficiency (Figure 4c), strongly suggesting that mitochondrial caspase-2 cleaved the substrate only when oxidative stress was induced.

### Loss of caspase-2 protects cells from mitochondrial oxidant-induced cell death

**Figure 5:**
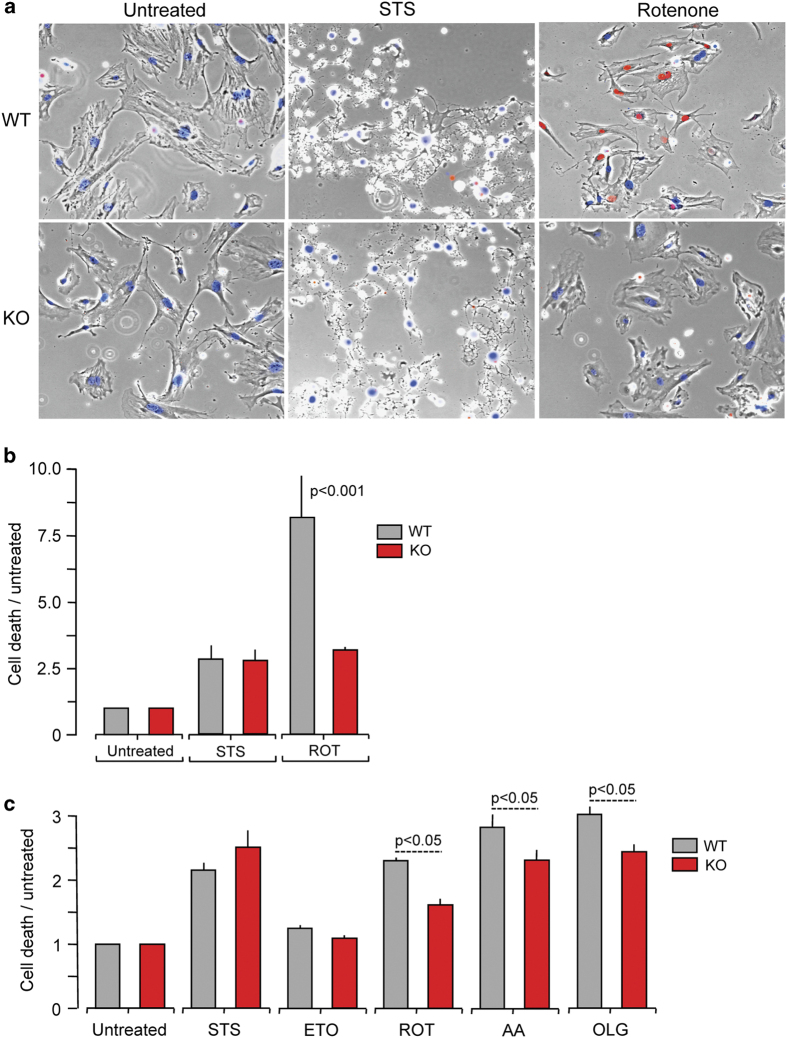
Loss of caspase-2 protects cells from mitochondrial oxidant-induced cell death. (**a**) Primary skin fibroblasts from newborn WT or *Casp2*^*−/−*^ mice on glass bottom chambers were treated with stressors as indicated (STS= 0.5 *μ*M staurosporine for 6 h; ROT=0.5 *μ*M rotenone for 30 h), stained with propidium iodide (PI) and Hoechst and imaged on a widefield microscope; representative photomicrographs are shown. (**b**) Percent PI-positive cells after normalization to total number of cells (Hoechst positive), followed by ratio to cell death in untreated cells. (**c**) Primary skin fibroblasts from newborn WT or *Casp2*^*−/−*^ mice were seeded in black bottom 96-well plates and treated with stressors as indicated (STS= 0.5 *μ*M staurosporine for 15 h; ETO=100 *μ*M etoposide for 24 h; ROT= 0.5 *μ*M rotenone for 30 h; AA= 10 *μ*M antimycin A for 24 h; OLG= 23 *μ*M oligomycin for 15 h). After treatment, cells were stained with PI and cell death levels were quantitated by measuring fluorescence on a plate reader. Digitonin was then added to lyse all cells and plates were read again. Percent cell death was calculated as the percent ratio of fluorescence intensities before and after digitonin treatment. Data represent values from three independent experiments. Data were analyzed using two-way ANOVA and Bonferroni post-tests.

Our data suggest that mitochondria contain caspase-2 that is activated by mitochondrial oxidants. A corollary to this observation is that loss of caspase-2 is associated with an alteration in cellular response to mitochondrial oxidant-induced apoptosis. Newborn skin fibroblasts isolated from WT and *Casp2*^*−/−*^ mice were treated with rotenone (mitochondrial complex I inhibitor), antimycin A (mitochondrial complex III inhibitor), oligomycin (mitochondrial complex V inhibitor), staurosporine (protein kinase inhibitor) and etoposide (topoisomerase II inhibitor). Cell death was detected with propidium iodide (PI) and apoptosis was quantified based on nuclear morphology (Figures 5a and b) observed using a confocal microscope. Apoptotic cell death was also monitored using a fluorescent plate reader (Figure 5c). Interestingly, while all treatments induced cell death in both WT and *Casp2*^*−/−*^ cells, the absence of caspase-2 significantly protected cells only from mitochondrial oxidant-induced cell death.

## Discussion

We have recently shown that absence of caspase-2 in intact live hepatocytes highly impairs the neutralization of mitochondrial reactive oxygen species^32^ (ROS), implying a critical role for caspase-2 in the mitochondria. Therefore, it is important to determine whether caspase-2 resides in the mitochondria and is active in this compartment. Previous reports have suggested that caspase-9,^18,33–35^ caspase-8,^18,36^ caspase-7,^18^ caspase-3,^18,33,35,37^ and also caspase-2^13,17,18^ localize to the mitochondria. However, the mitochondrial localization of caspase-2, has been questioned as other studies failed to detect mitochondrial caspase-2 using immunodetection approaches.^15,19,23^ Therefore, we used several different techniques to identify the presence of mitochondrial caspase-2.

Initially, we used a monoclonal antibody directed against caspase-2 to probe mitochondrial and cytosolic fractions from homogenized fibroblasts *in vitro* and from WT and *Casp2*^*−/−*^ liver *in vivo* using immunoblots (Figure 1a). We observed a relatively less intense band for caspase-2 in WT mitochondria as compared with cytoplasmic homogenates. Importantly, this band was absent in mitochondria isolated from *Casp2*^*−/−*^ mice indicating specificity of the antibody. We speculate that the low expression of caspase-2 in mitochondria correlates with the widely accepted dogma that apical caspases, and caspase-2 in particular, can self-activate without exogenous stimuli at higher concentrations. Therefore, the concentration of caspase-2 in mitochondria has to be tightly controlled, such that only a very small amount of activated caspase-2 would be sufficient to quickly initiate apoptosis when required. The use of different cell types, antibodies and detection reagents employed in our studies may perhaps explain why previous reports failed to detect caspase-2 in the mitochondria. Nevertheless, our experiments represent the first time that mitochondrial fractions from caspase-2-deficient tissues have been directly compared with mitochondrial fractions obtained from WT mice, and suggest that caspase-2 does exist in this compartment.

While we confirmed the purity of our subcellular fractions using organelle-specific markers, we also used immunofluorescence to detect caspase-2 in intact cells. We used a different monoclonal caspase-2 antibody and examined its colocalization to mitochondrial-specific proteins. Similar to western blotting, this technique also demonstrated that a subset of caspase-2 localized in the mitochondrial compartment. Since caspase-2 has been reported to be present in the ER,^11^ we speculated whether caspase-2 could be present at sites where ER and mitochondria are in contact. Hence, we determined colocalization of caspase-2 to the ER lumen-specific protein, CRT and as controls, the colocalization of CRT and the mitochondrial protein, AIF. Our results demonstrate that while caspase-2 is localized in the mitochondria, very little, if any, exists in the ER.

Importantly, FRET analyses using a mitochondrial DNA construct that expressed the caspase-2 substrate sequence, VDVAD, flanked by donor and acceptor fluorophores also indicated mitochondrial caspase-2 activity (Figure 4). We detected cleavage of our mitochondria-localized caspase-2 reporter molecule when cells were treated with the oxidant, *t*-BuOOH, but not with STS. We hypothesize that caspase-2 is not activated in mitochondria during STS-induced apoptosis; however, we cannot exclude the possibility that mitochondrial caspase-2 activation occurs at a time point beyond our measurements. While this technique takes advantage of the ability of assessing substrate cleavage in the mitochondria of intact cells, our experimental design also bears the caveat of using a somewhat non-specific substrate target sequence that can be cleaved by other caspases. Further analysis of our probe’s sensitivity to various conditions of cell stress would also help broaden the impact and utility of our approach. For example, changes in pH, oxidative stress and/or various levels of matrix Ca^2+^ are bound to affect the sensitivity of the probe. Hence, the generation of experimental data independent of FRET measurements can be very helpful in this regard.

Since our assays suggested that caspase-2 was active in the mitochondria, we next asked if mitochondrial caspase-2 could directly induce apoptosis or if cytosolic caspase-2 was first required to translocate to the mitochondrial compartment to induce apoptosis, as has been shown in *in vitro* studies.^21^ Towards this end, we employed a cell-free, mitochondrial reconstitution assay system in which mouse liver nuclei were monitored for the appearance of apoptotic morphology (Figure 2) and followed up with analysis of cytochrome *c* release (Figure 3). Our studies indicated that caspase-2 was present in the mitochondria and that mitochondrial caspase-2 could mediate signals that induced apoptotic changes in nuclear morphology in the absence of cytosolic caspase-2. The cell-free system approach combined with the use of a complete ablation of the caspase-2 gene allowed us to separate the contribution of both cytosolic and mitochondrial caspase-2 in modulating nuclear apoptotic morphology. Only when we reconstituted mitochondria from WT mice together with cytosol from *Casp2*^*−/−*^ mice did apoptotic nuclear morphology reach the levels observed using WT cytosol and mitochondria. These results indicate that caspase-2 is activated within the mitochondria and plays a critical role in apoptosis.

In congruence with the above data, we find that absence of caspase-2 in primary skin fibroblasts protects against cell death induced by electron transport chain inhibitors such as rotenone, antimycin A and oligomycin. Inhibition of complex I by rotenone and complex III by antimycin A in the mitochondrial electron transport chain is widely known to generate ROS in mitochondria and it has been speculated that these radicals are implicated in age-dependent degenerative diseases. Oligomycin can also produce free radicals under certain conditions.^38,39^ In addition, we find that lack of caspase-2 does not provide any protection against STS and etoposide. Our data are consistent with studies from other groups that have reported no difference in cell death when transfected with siRNA constructs to silence caspase-2 followed by exposure to etoposide.^40,41^

Interestingly, caspase-2 appears to contain a potential mitochondrial targeting sequence. A query of the amino acid sequence of murine caspase-2 on the iPSORT targeting prediction database^42^ based on the prediction by the κ-Nearest Neighbors Classifier algorithm,^43^ revealed the following probabilities of targeting peptides contained in caspase-2: 21.7% mitochondrial, 43.5% nuclear, 21.7% cytoplasmic, 8.7% vesicles of the secretory system and 4.3% vacuolar. A short mitochondrial targeting sequence is indicated at the amino terminus in mouse and rat, but not in human (Supplementary Figure S5). However, the human sequence has the same number of basic amino acid residues. Amino-terminal mitochondrial targeting sequences contain 15–30 basic amino acid residues. Since the predicted mitochondrial targeting sequence for caspase-2 is much shorter, it might be that only a very small percentage of caspase-2, or in certain circumstances, is shuttled to the mitochondria. This might provide an additional explanation on why it has been so difficult to detect caspase-2 in this compartment. Another possible reason for the low probability for caspase-2 to be directed to the mitochondria could be that only a particular isoform may be localized here, similar to isoforms of ornithine decarboxylase.^44^ Caspase-2 gene also contains more than one start site^45^ and produces several splice isoforms of mRNA;^46^ hence, a very small portion of caspase-2 might be targeted to the mitochondria, facilitating regulation at the genome level. Nonetheless, taken together, our data suggest that caspase-2 is present and active in the mitochondria.

## Materials and Methods

### Cell culture

WT and *Casp2*^*−/−*^ (KO) primary MEFs, skin fibroblasts from newborn mice and NIH/3T3 cells were cultured in DMEM supplemented with 10% FBS and antibiotics (Life Technologies, Carlsbad, CA, USA) at 37 °C in a humidified atmosphere of 5% CO_2_/95% air. Primary cells were not used beyond five passages.

### Immunocytochemistry

WT and *Casp2*^*−/−*^ MEFs were plated at a density of 2×10^4^ cells per well onto glass coverslips placed in 24-well plates. After overnight incubation, cells were fixed with 4% paraformaldehyde at room temperature (RT) for 20 min, washed, permeabilized with 0.3% Triton-X 100, blocked in 1% BSA for 1 h at RT and immunolabeled with primary antibodies: caspase-2 (clone 10C6; Enzo Life Sciences, Farmingdale, NY, USA); CRT, mitochondrial complex II (Abcam, Cambridge, MA, USA); AIF (Cell Signaling Technologies, Inc., Danvers, MA, USA) and MnSOD (Santa Cruz Biotechnology, Inc., Dallas, TX, USA). After incubation overnight at 4 °C, cells were washed with PBS containing 0.1% Tween-20 and incubated for 2 h at RT with appropriate flurorochrome-conjugated secondary antibodies (Life Technologies). Nuclei were stained with 10 *μ*g/ml Hoechst 33528 (Molecular Probes, Eugene, OR, USA). Coverslips were mounted with Vectashield (Vector Laboratories, Burlingame, CA, USA) onto slides and sealed. Approximately 15–20 cells were imaged on a confocal microscope (FV1000, Olympus, Center Valley, PA, USA) and scored blindly. Experiments were repeated three times and Pearson’s correlation coefficient was calculated using an Image J plugin (JaCoP) and analyzed for significance using ANOVA. Background subtraction was performed to eliminate noise. Spots analyzed were plotted as the normalized intensity of caspase-2 (green) as a function of mitochondria (red).

### Western blots

Protein samples were loaded onto 4–12% continuous SDS-PAGE gels (Life Technologies), transferred to PVDF membranes, blocked with 5% milk for 1 h and probed with the following primary antibodies at 4 °C overnight: caspase-2 (clone 11B4; Enzo Life Sciences), complex II (clone 21A11AE7; Abcam) and GAPDH (Cell Signaling Technology). After washing, blots were incubated with appropriate HRP-conjugated secondary antibodies and developed using a chemiluminescent system (SuperSignal West Pico or Femto, Thermo Fisher Scientific, Waltham, MA, USA).

### Isolation of mitochondrial and cytosolic fractions

Mitochondria and cytosol were obtained from WT or *Casp2*^*−/−*^ MEFs, liver and brain as described previously.^47^ Samples were homogenized on ice in a Dounce homogenizer containing isolation buffer (250 mM mannitol, 75 mM sucrose, 500 mM EGTA, 100 *μ*M EDTA and 10 mM HEPES) and centrifuged at 1000×*g* for 12 min at 4 °C. The supernatant was re-centrifuged to eliminate tissue and cell debris followed by further centrifugation at 10 000×*g* for 15 min at 4 °C to separate the mitochondrial and cytosolic fractions. Pelleted mitochondria was resuspended in isolation buffer containing 0.5% BSA, washed in a buffer comprising 250 mM mannitol, 75 mM sucrose, 100 *μ*M EDTA, 10 mM HEPES, 0.5% BSA) and finally resuspended in the above buffer but lacking BSA. Purity of mitochondrial preparation was determined by immunoblots.

### Cell-free apoptosis assays

Cell-free apoptosis assays were performed as described.^31^ Briefly, oocytes were surgically removed from *Xenopus laevis*, defolliculated with collagenase type IV (Sigma-Aldrich, , St. Louis, MO, USA), rinsed three times with egg lysis buffer (250 mM sucrose, 50 mM KCl, 2.5 mM MgCl_2_, 1 mM DTT, 20 mM HEPES-KOH; 50 mg/ml cycloheximide; 5 mg/ml cytochalasin B, 10 mg/ml pepstatin; 10 mg/ml leupeptin; pH 7.5) and centrifuged at 2000 r.p.m. for 2 min at 4 °C. The supernatant phases were pooled, transferred to Beckman Polyallomer tubes (Beckman Coulter, Inc., Indianapolis, IN, USA), overlayed with mineral oil and centrifuged twice at 10 000×*g* for 12 min at 4 °C. The resulting supernatant fraction was transferred to fresh tubes and ultracentrifuged at 200 000×*g* for 1 h at 4 °C to obtain cytosol and mitochondrial fractions. Rat liver nuclei were incubated with either cytosol alone, or cytosol with mitochondria isolated from WT or *Casp2*^*−/−*^ brain or liver. In other experiments, WT or *Casp2*^*−/−*^ liver was fractionated to yield cytosolic, mitochondrial and nuclear fractions and mix-and-match experiments were performed. All samples were incubated with a mixture of creatine phosphate, creatine phosphokinase (Calbiochem, Billerica, MA, USA) and dATP (EMD Chemicals, Gibbstown, NJ, USA). Reactions were carried out at 37 °C for different experimental time periods as indicated. Nuclei were stained with 10 *μ*g/ml Hoechst 33528 (Molecular Probes) and samples were transferred to glass slides and apoptotic features were determined using a widefield TE200 Nikon Eclipse inverted microscope with a ×60 water objective. Images were acquired using standard UV filters, collected with Open Lab software (Improvision, Lexington, MA, USA) and deconvoluted for maximum clarity. A minimum of 10 fields per sample and 10–20 cells per field was analyzed.

### Release of cytochrome *c*

Purified *Xenopus* oocyte cytosol was incubated with purified WT or *Casp2*^*−/−*^ brain mitochondrial fractions at 37 °C for 2 or 4 h; nuclei were not added. Samples were then centrifuged for 5 min at 12 000×*g* to separate mitochondrial and cytosol fractions. The mitochondrial pellet was resuspended and probed on immunoblots for cytochrome *c* (BD Biosciences, San Jose, CA, USA) or actin (Santa Cruz Biotechnology, Inc.).

### FRET analyses

Mitochondrial Casp2 FRET construct, mC2Y, was generated by subcloning the mitochondrial targeting sequence from subunit VIII of human cytochrome *c* oxidase using pCMV/mito vector^48^ (Life Technologies) and an enhanced CFP fragment (pECFP-N1; Clontech Laboratories, Inc., Mountain View, CA, USA), linked to the 5-prime end of the preferential caspase-2 substrate (VDVAD) coding sequence, into the mammalian expression vector pEYFP-N1 (Clontech Laboratories, Inc.). Specifically, the protein sequence of the linker construct joining ECFP and EYFP is AVDVADARDPPVAT. As a non-substrate control, the caspase-2 substrate coding sequence was replaced by a sequence coding for a string of glycine residues of equal length to generate mCGY. All constructs were sequenced before use. For experiments, 6×10^4^ NIH/3T3 cells were seeded onto glass coverslips in a six-well plate and transfected with FRET constructs using FuGene 6 (Roche, Indianapolis, IN, USA). After 24 h, apoptosis was induced with 0.5 μM staurosporine (STS) (Enzo Life Sciences) or 100 μM *tert*-butyl-hydroperoxide (t-BOOH) (Sigma-Aldrich) for 6 h. Both treatments were performed in complete medium. Following treatment, cells were rinsed with PHEM buffer (60 mM PIPES, 25 mM HEPES, 10 mM EGTA, 2 mM MgCl_2_, pH 7.3), fixed with 4% paraformaldehyde and incubated with sodium borohydride (1 mg/ml) to reduce autofluorescence. Coverslips were mounted onto microscope slides using Vectashield (Vector Laboratories, Inc.) and sealed. Acceptor photobleaching was performed using a Zeiss 510 LSM inverted confocal microscope (Carl Zeiss Microimaging, Thornwood, NY, USA). A ×60 immersion oil objective was used along with the following filters: for CFP, BP470-500 emission, HFT458/514 dichroic beam splitter and 458 excitation filters; for YFP, BP550/50 emission, HFT458/514 dichroic beam splitter and 514/10 emission filters. Differences in CFP fluorescence intensity was measured in mitochondria and FRET efficiency was calculated using the equation *E*=(1−IDA)/ID, where IDA is the intensity of CFP before YFP photobleaching and ID is the intensity of CFP after photobleaching. In all, 5–10 regions were analyzed within one cell, a minimum of 10 cells were analyzed per experiment and each experiment was repeated three times.

### Cell death assays

Primary skin fibroblasts from newborn WT or *Casp2*^*−/−*^ mice were plated at 6×10^3^ cells per well onto two-well glass bottom chambers overnight and treated with 0.5 *μ*M staurosporine for 6 h or 0.5 *μ*M rotenone for 30 h, stained with 0.02 mg/ml PI and 5 *μ*g/ml Hoechst 33258, for 20 min in dark and visualized under a widefield microscope. Bright and fluorescent field photomicrographs were taken and superimposed to count total and PI-positive cells. Percent PI-positive cells were determined. Alternatively, 8×10^4^ cells were seeded in black bottom 96-well plates overnight and treated with different stressors: 0.5 *μ*M staurosporine for 15 h; 100 *μ*M etoposide for 24 h; 0.5 *μ*M rotenone for 30 h; 10 *μ*M antimycin A for 24 h or 23 *μ*M oligomycin for 15 h. After treatment, cells were stained with 2.5 *μ*M PI and cell death levels were quantitated by measuring fluorescence on a plate reader (Perkin Elmer, Waltham, MA, USA) using 540 nm excitation and 620 nm emission filters. Digitonin (0.25 mg/ml) was then added to gently lyse all cells and plates were read again. Percent cell death was calculated as the percent ratio of fluorescence intensities before and after digitonin treatment.

### Statistical analysis

Experimental results were statistically analyzed either using one-way or two-way ANOVA with Bonferroni post-test or a Student’s *t*-test using GraphPad Prism ver 5.0 (GraphPad, La Jolla, CA, USA).
