# Supplementary figures and images for: Caspase-2 resides in the mitochondria and mediates apoptosis directly from the mitochondrial compartment
Source: Cell Death Discov. 2016 Feb 15;2:16005–. doi: 10.1038/cddiscovery.2016.5 (PMC4806400; doi:10.1038/cddiscovery.2016.5)

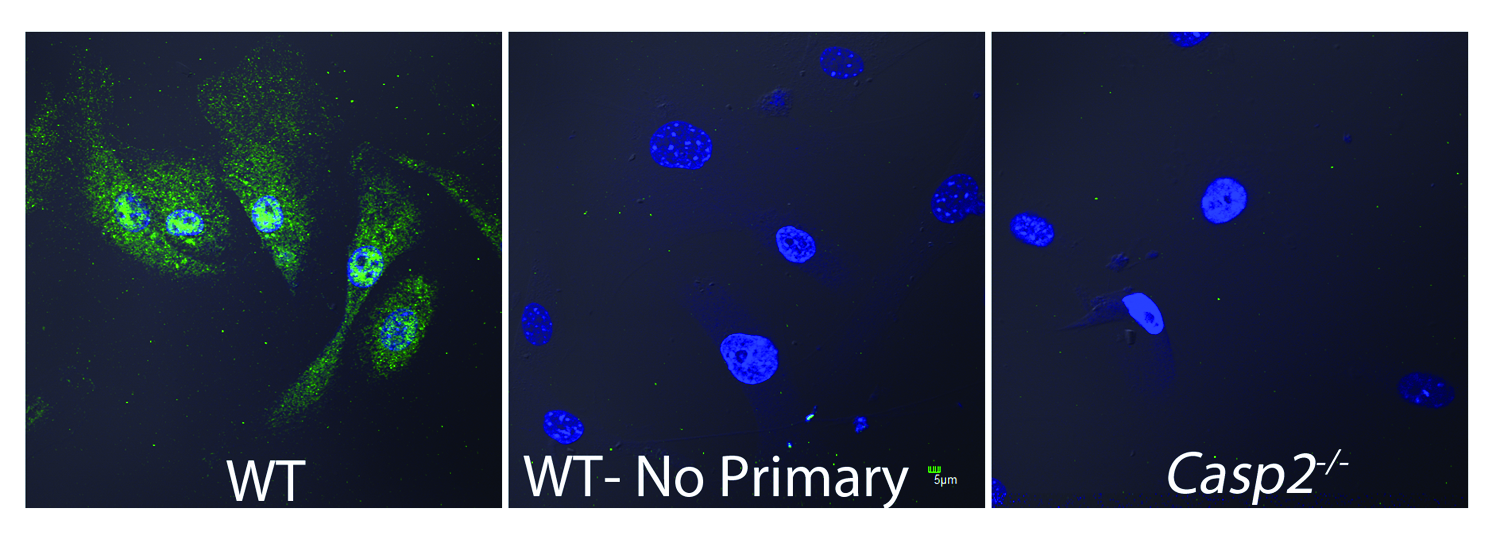

Supplement: Supplementary Figure 1 [file cddiscovery20165-s1.tiff]

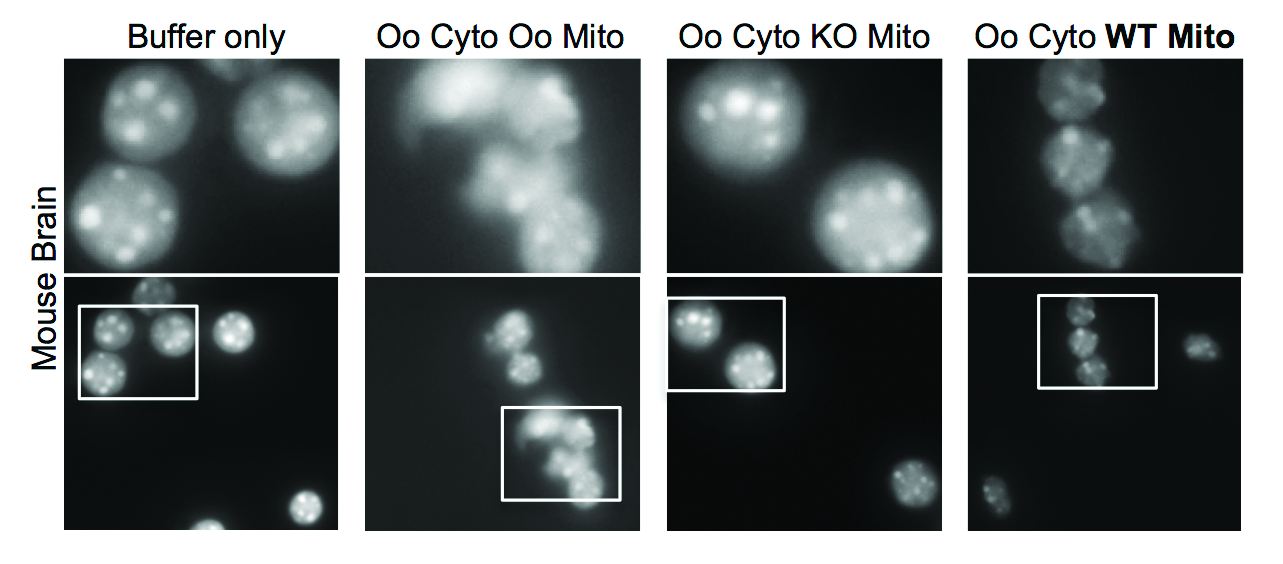

Supplement: Supplementary Figure 2 [file cddiscovery20165-s2.tiff]

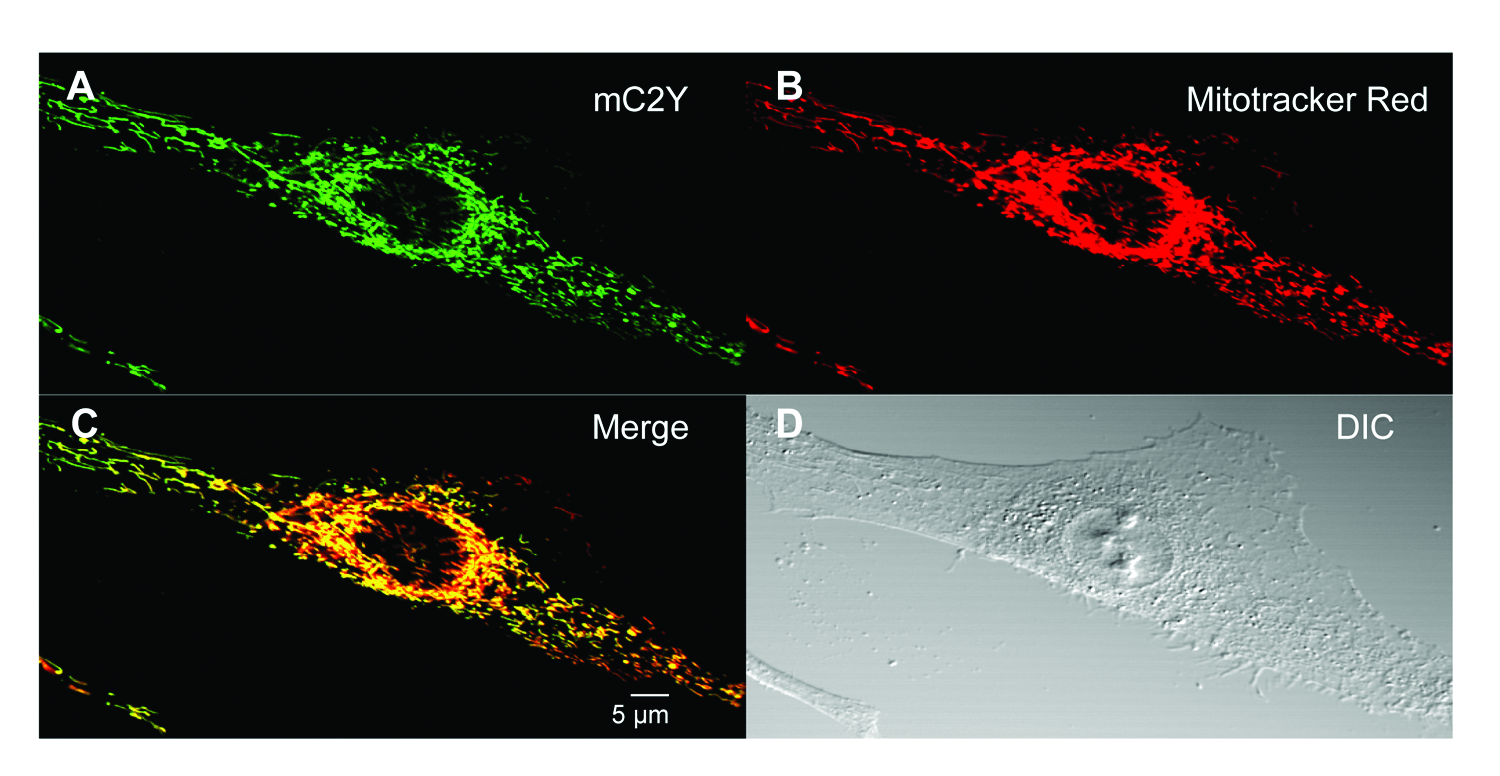

Supplement: Supplementary Figure 3 [file cddiscovery20165-s3.tiff]

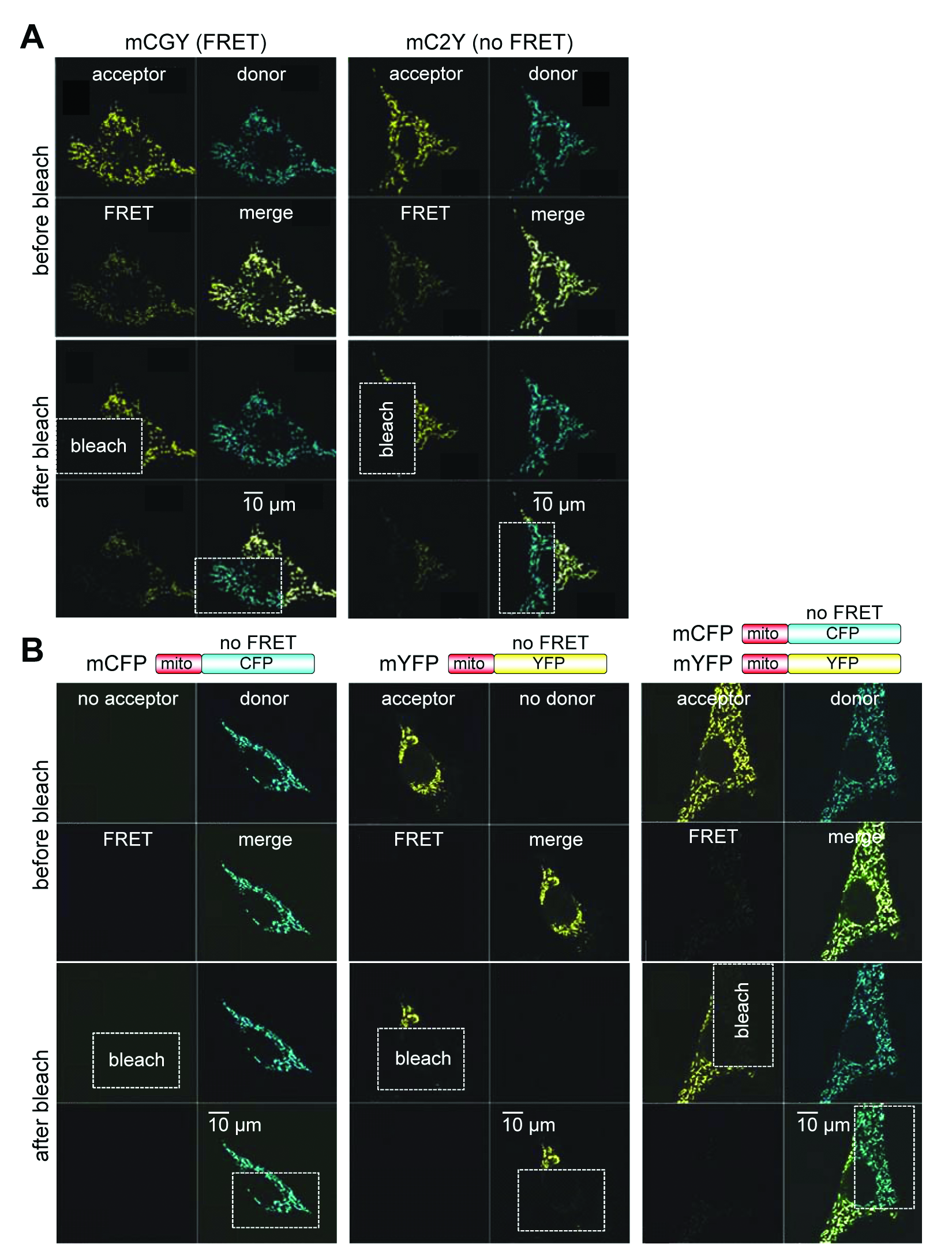

Supplement: Supplementary Figure 4 [file cddiscovery20165-s4.tiff]

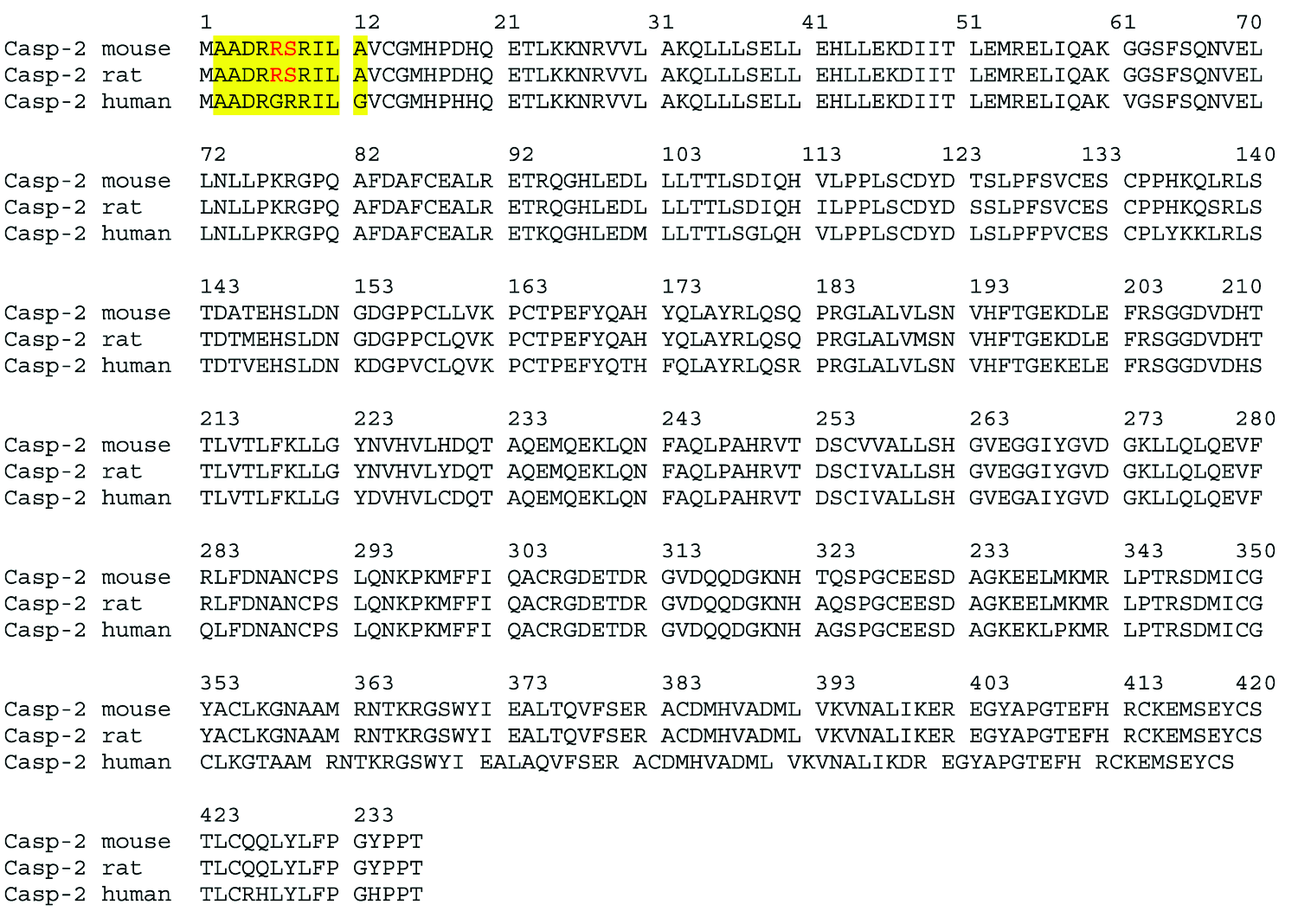

Supplement: Supplementary Figure 5 [file cddiscovery20165-s5.tiff]
